# Efficacy of *Streptomyces melanosporofaciens* strain X216 at controlling clubroot disease on oilseed rape

**DOI:** 10.3389/fmicb.2023.1249813

**Published:** 2023-09-19

**Authors:** Lin Ding, Hu Zhou, Hai-di Liang, Lin Tan, Hui Zhao, Xiao-jun Chen, Zuo-hua Ren

**Affiliations:** ^1^College of Plant Protection, Hunan Agricultural University, Changsha, China; ^2^Yueyang Inspection and Testing Center, Yueyang, China; ^3^College of Agricultural, Hunan Agricultural University, Changsha, China

**Keywords:** actinomycete, clubroot, *Plasmodiophora brassicae*, resting spore, biological control

## Abstract

Oilseed rape (*Brassica napus* L.) is highly susceptible to infection from the soilborne pathogen *Plasmodiophora brassicae* Woronin that causes clubroot disease and deleteriously affects production throughout the world. In this study, biological control resources were explored by isolating 237 strains of bacteria from fields of oilseed rape using the gradient dilution coating method. A strain with strong antagonistic ability was screened using a plate confrontation test and designated X216. It was identified as *Streptomyces melanosporofaciens* owing to its morphological characteristics and 16S rRNA gene sequence. This study also examined the lethality of strain X216 to the resting spores of *P. brassicae*, its influence on infection in root hairs, and its ability to control clubroot on oilseed rape. The corrected lethality rate on resting spores after strain X216 had been used for 14 days was 56.59% ± 1.97%, which was significantly higher than the use of 75% of the fungicides chlorothalonil WP and 20% Fluazinam SC. Significantly fewer root hairs were infected after this treatment. A pot test showed that X216 was 62.14% effective at controlling the disease, which was not significantly different from that of the fungicide 100 g L^−1^ cyazofamid SC diluted 1,000-fold but significantly higher than those of 75% chlorothalonil and 50% carbendazim WP. Strain X216 controlled 43.16% of the incidence of clubroot in the field, which could significantly reduce the disease index of oilseed rape clubroot. Therefore, strain X216 is promising to study for the biological control of oilseed rape clubroot.

## Highlights

- *Streptomyces melanosporofaciens* X216 inhibited cotton fusarium wilt pathogen *F. oxysporum*.
- *Streptomyces melanosporofaciens* X216 can be lethal to resting spores of *P. brassicae*.
- *Streptomyces melanosporofaciens* X216 can control oilseed rape clubroot in greenhouse pots and the filed.

## 1. Introduction

Clubroot disease, caused by the obligate parasitic pathogen *Plasmodiophora brassicae* Woronin, is considered to be a major devastating disease of cruciferous plants (Botero et al., 2019). It results in the serious reduction of the production of cruciferous vegetable crops throughout the world. Moreover, the resting spores of *P. brassicae* can survive for up to 20 years in soil, which makes it difficult to prevent and treat (Schwelm and Ludwig-Müller, 2021). The lifecycle of *P. brassicae* begins in the soil. Resting spores are more likely to germinate if the host is present in the soil because the root exudates of the host plant facilitate the germination of *P. brassica* (Pérez-López et al., 2020). After infection with this pathogen, the gradual formation of clubbed roots will limit the absorption of water and nutrients by the plants, which leads to wilting, the retardation of growth, and even the death of infected plants in severe cases (Kageyama and Asano, 2009).

Traditional methods of breeding disease-resistant varieties to control clubroot disease are generally ineffective. Currently, the prevention and control of clubroot disease primarily relies on chemical fungicides, but their enormous use has not eradicated clubroot disease (Ahmed et al., 2020). To reduce the environmental pollution caused by the use of chemical fungicides to control clubroot disease, researchers are consistently exploring biological resources to control clubroot disease. For example, Hu et al. (2021) studied *Streptomyces alfalfae* XY25^T^ as a biological control agent, and the bacteria controlled clubroot disease by 69.4%. Zhao et al. (2022) showed that *Trichoderma* Hz36 controlled clubroot disease on oilseed rape and Arabidopsis by 44.29 and 52.18%, respectively. Jäschke et al. (2010) showed that the endophytic fungus *Acremonium alternatum* reduced the disease index of clubroot disease by 50% and limited the formation of clubbed roots. Liu et al. (2018) isolated *Bacillus subtilis* XF-1 from the rhizosphere of Chinese cabbage (*Brassica napus* subsp. *pekinensis*), which controlled clubroot disease by 76.92%.

In this study, an antagonistic microorganism was screened from fields of oilseed rape (*B. napus*) and identified by morphology and molecular biology. Its ability to control clubroot in oilseed rape was determined by its lethality to resting spores, influence on the rate of infection by root hairs, and ability to control this disease in pot and field tests, which explored resources for the biological control of clubroot disease.

## 2. Materials and methods

### 2.1. Test materials

Microorganisms were isolated from soil that surrounded healthy plants in oilseed rape fields that contained plants infected with clubroot disease. Roots that exhibited symptoms of clubroot disease were collected from Hengyang County, Hengyang City, Hunan Province, China (N27°06′57.14″, E112°24′54.85″), and identified as infected with pathotype 4 of *P. brassicae* after cleaning. The roots were stored at −20°C for further use after they had been cleaned.

The culture media that were used included Gause’s Synthetic Agar Medium, Gause’s Synthetic Broth Medium, and potato dextrose agar (PDA). Liangyou No. 9 (Hengyang Vegetable Seed Limited Company, Hengyang, China) was the variety of oilseed rape that was used in this study. It is highly susceptibility to clubroot disease. The reagents used in this study included Evans blue, aniline blue, Hoagland modified nutrient salts solution, and calcium oxide (CaO). The fungicides included 100 g L^−1^ cyazofamid SC, 20% fluazinam SC, 75% chlorothalonil, and 50% carbendazim WP.

### 2.2. Plate screening of biocontrol actinomycetes

A hole punch (d = 5 mm) was used to punch holes in a plate of activated cotton Fusarium wilt pathogen (*Fusarium oxysporum* f. sp. *vasinfectum*), and the pathogen cake was inoculated in the center of a Gause’s Synthetic Agar Medium plate. Simultaneously, the pathogen cake was symmetrically inoculated with different microorganisms that had been isolated from the soil. Each strain was inoculated three times, and the plates were inverted and cultured in a constant temperature incubator at 28°C for 5 days. The formation of an inhibition zone was monitored.

A secondary screening was conducted using the plate confrontation method (Zhang H. et al., 2022) to determine the antagonistic effect of the strains that had been screened initially against the causal agents of cotton fusarium wilt, strawberry blight (*Phytophthera fragariae*), strawberry root rot (*Fusarium oxysporum*), and northern corn leaf blight (*Exserohilum turcicum*).

The pathogen cake (d = 5 mm) was inoculated in the center of a Gause’s Synthetic Agar Medium plate, and the strain to be tested was inoculated on both sides of the pathogen cake at the same distance from the pathogen cake and the edge of plate. A plate that was only inoculated with the pathogen cake was used as the control. There were six replicates for each treatment, and after 7 days of inverted culture at 28°C in a constant temperature incubator, the inhibition rate was calculated.


$$\begin{array}{l} \mathrm{Inhibition}\;\mathrm{rate}\;\left(\%\right)=[(\mathrm{growth}\;\mathrm{diameter}\;\mathrm{of}\;\mathrm{control}\;\mathrm{colony}-\\ \;\mathrm{growth}\;\mathrm{diameter}\;\mathrm{of}\;\mathrm{treatment}\;\mathrm{colony})/\\ \;\mathrm{growth}\;\mathrm{diameter}\;\mathrm{of}\;\mathrm{control}\;\mathrm{colony}]\times 100. \end{array}$$

### 2.3. Identification of biocontrol actinomycetes

#### 2.3.1. Morphological identification

The mycelia of the strain were inoculated on Gause’s Synthetic Agar Medium plates and potato dextrose agar (PDA) plates, respectively, and then incubated at 28°C in a constant temperature incubator for 7–14 days. The growth rate of the strain and its colony morphology, color, size, and other characteristics, as well as whether it produces soluble pigments and the color of any soluble pigments, were continuously observed and recorded. The strain was cultured using the coverslip culture method, and morphological characteristics, such as the mycelia and chains of spores, were observed under an electron microscope after 14 days (Tang et al., 2023).

#### 2.3.2. Molecular biological identification

A TransGen *EasyPure*^®^ Genomic DNA Kit was used to extract DNA from the actinomycetes (TransGen, Beijing, China). The bacterial universal primers 27F (5’-AGAGTTTGATCCTGGC TCAG-3′) and 1492R (5′- GGTTACCTTGTTACGACTT-3′) were used to amplify the 16S rRNA gene sequence using PCR (Li X. et al., 2021). The amplified fragment was approximately 1500 bp. The PCR reaction system (20 μL) included 2 μL DNA template, 10 μL Taq PCR SuperMix, 6 μL ddH_2_O, and 1 μL each of the 27F and 1492R primers. The PCR reaction program was as follows: pre-denaturation at 94°C for 5 min, denaturation at 94°C for 30 s, annealing at 55°C for 30 s, extension at 72°C for 1 min for a total of 34 cycles, and a final extension at 72°C for 1 min (Hamid et al., 2020). The PCR amplification product was checked using 1% agarose gel electrophoresis, and the remaining product was sent to Sangon Biotech (Shanghai, China) for sequencing. The homology of the sequenced sequences was compared on the NCBI website to obtain the sequences of related species, and a phylogenetic tree was constructed using MEGA.

### 2.4. Preparation of a resting spore suspension of *Plasmodiophora brassicae*

The stored clubbed roots were defrosted and incubated at 25°C in the dark for 5 days until they had decayed. The roots were crushed with a pulverizer. Sterile water was added and stirred well, the homogenate was then filtered through eight layers of gauze and filtered in a centrifuge at 500 rpm for 15 min. The precipitate was discarded, and the supernatant was collected. Centrifuge tubes of 50 mL were centrifuged at 2500 rpm for 5 min. The precipitate was collected and centrifuged three to four times more to obtain more resting spores, disinfected with 2% (v/v) chloramine T for 20 min, centrifuged at 3000 rpm for 5 min to collect the precipitate, suspended with sterile water, and centrifuged three times. The precipitate was suspended in sterile water and treated with 1 μg mL^−1^ colistin sulfate, 1 μg mL^−1^ vancomycin hydrochloride and 6 μg mL^−1^cefotaxime sodium, respectively (Asano et al., 1999; Luo et al., 2014). The precipitate was placed at 24°C in the dark for 24 h and centrifuged at 3000 rpm for 5 min to collect the precipitate. Sterile water was added to suspend the precipitate, which was then centrifuged at 3000 rpm for 5 min. This process was repeated three times. The concentration of resting spores was calculated using a blood cell counting plate. Finally, the suspension of resting spores was adjusted to 1 × 10^8^ mL^−1^ with sterile water and stored at 4°C for later use.

### 2.5. Preparation of oilseed rape root secretions

Oilseed rape seeds were soaked in 70% (v/v) alcohol for 30 s, washed with sterile water, treated with 5% (v/v) NaClO for 5 min, and finally rinsed three times with sterile water. The seeds were planted in sterile petri dishes covered with filter paper, and the filter paper was soaked with 5 mL of sterile water. The tops of the petri dishes were sealed with plastic wrap to keep them moist. The seeds were incubated in a constant temperature incubator at 25°C, and the seedlings were transplanted after the cotyledon had unrolled. The seedlings were transplanted into a centrifuge tube that contained Hoagland nutrient solution (Huang et al., 2022) with cotton and cultured for 7 d at 25°C in an incubator with a light/dark cycle of 12 h/12 h. The root secretions were filtered through eight layers of gauze and then filtered through a microporous filter membrane with a pore size of 0.22 μm to remove any contaminating bacteria before storage at 4°C for later use.

### 2.6. Preparation of the actinomycete culture solution

Gause’s Synthetic Broth Medium was used to prepare the culture media for actinomycetes. After the actinomycete was activated on Gauze’s Synthetic Agar Medium for 7 d, holes were punched with a 5 mm diameter punch. A volume of 150 mL of Gause’s Synthetic Broth Medium was added to 300 mL conical bottles, and 15 pathogen cakes were placed in each conical bottle. The bottles were cultured at 28°C in a shaker for 7 d with a rotation speed of 180 rpm. The culture solution obtained was filtered through eight layers of gauze and then by a 0.22 μm microporous membrane before storage at 4°C for future use.

### 2.7. Lethal effect of actinomycetes and different fungicides on the resting spores of *Plasmodiophora brassicae*

#### 2.7.1. Lethal effect of actinomycete on the resting spores of *Plasmodiophora brassicae*

Centrifuge tubes that held 10 mL were used as the container, and 4 mL of reaction solution was added. The concentration of sterile culture solution of actinomycetes was diluted 1-, 2-, 5-, 10-, and 20-fold with sterile water. The root secretions were used to dilute the suspension of resting spores, and the final concentration was 1 × 10^7^ spores mL^−1^. In the experimental group, 2 mL of resting spore suspension and 2 mL of actinomycete sterile filtrate of different concentrations were added to the centrifuge tube, respectively. In the control group, 2 mL of resting spore suspension and 2 mL of sterile water were added, and this was repeated three times. The solution was shaken daily with an oscillator to help resuspend the spores and incubated in the dark at 25°C.

#### 2.7.2. Lethal effect of different pesticides on the resting spores of *Plasmodiophora brassicae*

Centrifuge tubes that held 10 mL were used as the container, and 5 L of reaction solution was added. The root secretions were used to dilute the resting spore suspension, and the final concentration was 1 × 10^7^ spores mL^−1^. A total of 100 g/L cyazofamid SC, 20% fluazinam SC, 75% chlorothalonil WP and 50% carbendazim WP were added to the resting spore suspension, respectively, using the minimum dosage of 5 mL calculated from the instructions. No fungicides were added to the control group. Each treatment was repeated three times. The spores were homogenized daily using an oscillator and incubated at 25°C in the dark.

#### 2.7.3. Method for testing the activity of *Plasmodiophora brassicae* resting spores

The activity of resting spores was observed microscopically after 7, 10, and 14 d, and the spores were stained with 2% (v/v) Evans blue solution, which was found to be superior to other types of stains. The resting spore suspension and 2% (v/v) of Evans blue were mixed well at 2:1 (v/v) and stained at room temperature for 24 h (Wang et al., 2022). A volume of 10 μL was pipetted to prepare temporary slides to observe the spores under a light microscope. The activity of resting spores was evaluated based on the extent to which the resting spores were stained. Resting spores that had lost their activity stained dark blue, and resting spores were active were not stained blue. A total of 100 dormant spores were observed at a time with three replicates.


$$\begin{array}{l} \mathrm{Resting}\;\mathrm{spore}\;\mathrm{mortality}=[\mathrm{number}\;\mathrm{of}\;\mathrm{dark}\;\mathrm{blue}\;\mathrm{spores}\;\mathrm{in}\\ \;\mathrm{the}\;\mathrm{field}\;\mathrm{of}\;\mathrm{view}/\;(\mathrm{number}\;\mathrm{of}\;\mathrm{dark}\;\mathrm{blue}\;\mathrm{spores}\;\mathrm{in}\;\\ \;\mathrm{the}\;\mathrm{field}\;\mathrm{of}\;\mathrm{view}\;+\;\mathrm{number}\;\mathrm{of}\;\mathrm{unstained}\;\mathrm{spores}\;\mathrm{in}\;\\ \;\mathrm{the}\;\mathrm{field}\;\mathrm{of}\;\mathrm{view})]\times 100\% \end{array}$$

$$\begin{array}{l} \mathrm{corrected}\;\mathrm{resting}\;\mathrm{spore}\;\mathrm{mortality}\;=\\ \;\mathrm{treated}\;\mathrm{spore}\;\mathrm{mortality}-\mathrm{control}\;\mathrm{spore}\;\mathrm{mortality} \end{array}$$

### 2.8. Effect of actinomycetes and different fungicides on the primary infection of *Plasmodiophora brassicae*

#### 2.8.1. Indoor hydroponic inoculation of *Plasmodiophora brassicae*

The resting spores of *P. brassicae* were diluted to 1 × 10^5^ spores mL^−1^ (Zahr et al., 2022) with Hoagland’s nutrient solution, and 10 mL was added to each 10 mL centrifuge tube. The concentration of actinomycete sterile filtrate was adjusted, so that it was diluted by 1-, 2-, and 5-fold in the resting spore suspension. A total of 100 g L^−1^ cyazofamid SC, 20% fluazinam SC, 75% chlorothalonil WP and 50% carbendazim WP were added to the resting spore suspension, respectively, using the minimum dosage of 10 mL calculated from the instructions. Each treatment was repeated 15 times, and no actinomycete solution or fungicides were add to the control group. Two seedlings were clamped to a 15 mm diameter cotton set with small cuts and fixed in centrifuge tubes, and each tube was kept dark by being wrapped with aluminum foil or black tape. The seedlings were placed in 25°C incubator and incubated at a light/dark cycle of 12 h/12 h. Adequate amounts of Hoagland’s nutrient solution were added each day to maintain the liquid in the centrifuge tube at a volume of 10 mL.

#### 2.8.2. Observation of *Plasmodiophora brassicae* infection of host root hairs

The root hairs were observed by microscopy at 3, 7, and 10 d after inoculation. Four seedling roots were sampled for each treatment period, and the seedlings were first rinsed with sterile water to remove some of the spores attached to the roots and then stained for 1–2 min with 125 mg L^−1^ of aniline blue prepared with 50% acetic acid (Sun et al., 2022). They were washed with sterile water to remove the floating color on the surface of the seedling roots. The infection rate was then calculated. A total of 100 root hairs were observed each time, and the procedure was repeated three times.


$$\begin{array}{l} \mathrm{Infection}\;\mathrm{rate}\;\mathrm{of}\;\mathrm{root}\;\mathrm{hairs}=\\ \;\left(\mathrm{number}\;\mathrm{of}\;\mathrm{infected}\;\mathrm{root}\;\mathrm{hairs}/\mathrm{total}\;\mathrm{observed}\;\mathrm{root}\;\mathrm{hairs}\right)\times 100\% \end{array}$$

### 2.9. Efficacy of actinomycetes and different fungicides on oilseed rape clubroot disease

#### 2.9.1. Efficacy in a pot test

The mycorrhizal soil method was used to inoculate *P. brassicae* with 1.0 × 10^8^ resting spores g^−1^ of soil. The water content of the soil was evaluated by hand by pinching the soil to ensure that it was comparable to bread dough and would disperse when pinched. They were sealed at 25°C for 48 h before use. The actinomycetes filtrate that was used to treat the roots was diluted 1-, 2-, and 5-fold. A total of 100 g L^−1^ of cyazofamid SC was diluted 1,000-, 1,500-, and 2,000-fold; 75% chlorothalonil WP was diluted 500-, 1,000-, and 1,500-fold, and 50% carbendazim WP was diluted 600-, 800-, and 1,000-fold. Calcium oxide (CaO) was added in increments of 1.2 g, 1.4 g, and 1.6 g kg^−1^ of soil. Water was used as the control treatment. There were 16 groups, and each group consisted of six pots. There were 10 oilseed rape seedlings per pot. With the exception of CaO treatment, each pot was irrigated with 100 mL of the different treatment groups at the time of sowing and 7 and 14 d later. The greenhouse was maintained at 25°C and received 12 h of light per day. The incidence of clubroot disease was investigated 45 d after the oilseed rape seedlings had emerged.

#### 2.9.2. Efficacy in the field test

The experimental field was located in Daguang Village, Hengyang County, Hengyang City, Hunan Province, China, and was part of a multi-year study on oilseed rape clubroot disease. The experiment was conducted in a randomized group design，planting 15 rapeseed plants in each hole of the field ridge (30 × 30 cm), 4 holes per row, two rows of one treatment, and three replicates, which were all managed in the same manner (Li J. et al., 2021). The 16 groups were the same as those described in Section 2.9.1. The CaO treatment involved its placement into the holes in advance with the soil mixed at different concentrations. The other treatment groups were irrigated with 500 mL of the different treatments per hole at the time of sowing and 14 d later. The plants were initially surveyed for the incidence of clubroot disease 45 d after the seedlings had emerged and after the control group had become infected.

#### 2.9.3. Criteria used to determine the disease intensity and quantification of the effectiveness of preventative treatments

The criteria used to classify the disease were as follows (NY/T, 3621-2020, Agricultural industry standard of the People's Republic of China, 2020): Grade 0, normal root system, asymptomatic; Grade 1, small clubbed roots <0.5 cm in diameter formed on one-third or fewer lateral roots, no clubbing on the main root; Grade 2, one-third to two-third of the lateral roots had clubbing, or the diameter or length of clubbing on the main root <3 times the diameter of stem base; Grade 3, two-third or more of the lateral roots exhibited clubbing, which was obvious on the main root, and the diameter or length of clubbing >3 times the diameter of stem base, or the clubbed roots were ulcerated.


$$\begin{array}{l} \mathrm{Disease}\;\mathrm{incidence}=\mathrm{number}\;\mathrm{of}\;\mathrm{diseased}\;\mathrm{plants}\\ \;/\mathrm{total}\;\mathrm{number}\;\mathrm{of}\;\mathrm{plants}\;\mathrm{surveyed}\times 100\% \end{array}$$

$$\begin{array}{l} \mathrm{disease}\;\mathrm{index}=\sum (\mathrm{disease}\;\mathrm{grade}\;\mathrm{representative}\;\mathrm{value}\\ \;\times \mathrm{number}\;\mathrm{of}\;\mathrm{diseased}\;\mathrm{plants}\;\mathrm{at}\;\mathrm{each}\;\mathrm{grade})\\ \;/(\mathrm{total}\;\mathrm{number}\;\mathrm{of}\;\mathrm{plants}\times \mathrm{highest}\;\mathrm{grade}\;\\ \;\mathrm{disease}\;\mathrm{representative}\;\mathrm{value})\times 100 \end{array}$$

$$\begin{array}{l} \mathrm{control}\;\mathrm{efficiency}=[(\mathrm{control}\;\mathrm{disease}\;\mathrm{index}\\ \;-\mathrm{treatment}\;\mathrm{disease}\;\mathrm{index})\\ \;/\mathrm{control}\;\mathrm{disease}\;\mathrm{index}]\times 100\% \end{array}$$

## 3. Results and analysis

### 3.1. Plate screening of biocontrol actinomycetes

**Figure 1:**
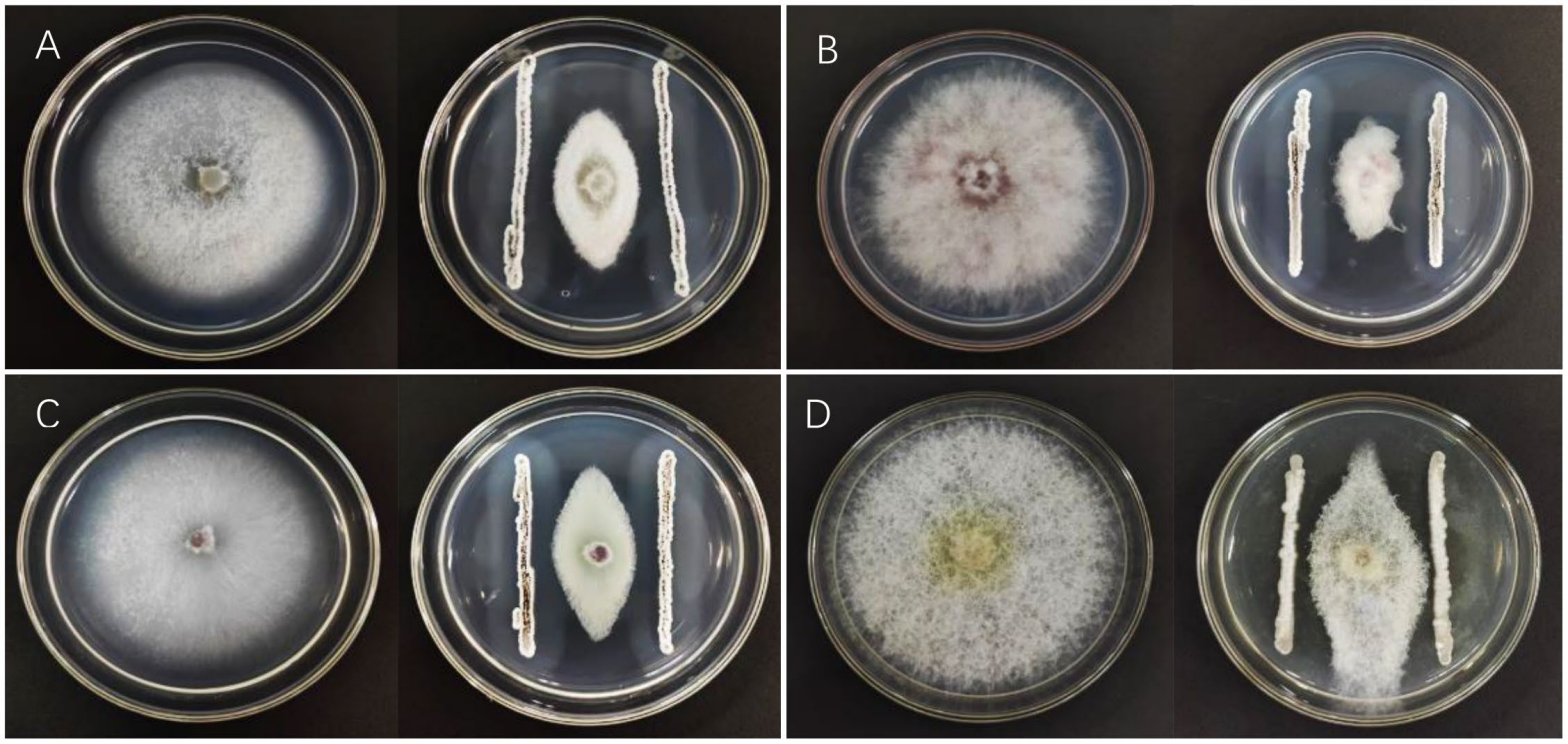
Inhibition of pathogens by *Streptomyces melanosporofaciens* X216. **(A)** Fusarium oxysporum **(B)** Phytophthera fragariae **(C)** Fusarium oxysporum **(D)** Exserohilum turcicum.

A total of 237 strains were isolated from the soil using the plate dilution method. Since *P. brassicae* cannot be cultured artificially, the pathogen *F. oxysporum* f. sp. *vasinfectum*, the causal agent of cotton fusarium wilt, was used for this experiment. This pathogen was selected as the indicator pathogen for plate screening in this study because it has cell walls that have a similar composition to those of *P. brassicae* (Zhu et al., 2020). The strain that was the most effective antagonist was selected for re-screening after the initial screening and designated X216. It inhibited *F. oxysporum* f. sp. *vasinfectum* by 63.84%, *Ph. fragariae* by 71.19%, the strawberry root rot pathogen by 70.76%, and *E. turcicum* by 51.87% (Figure 1; Table 1).

**Table 1:** Rate of inhibition of *Streptomyces melanosporofaciens* X216 against different pathogens.

Pathogen	Inhibition rate/%
Fusarium wilt of cotton (*Fusarium oxysporum*)	(63.84 ± 1.35)b
Strawberry blight (*Phytophthera fragariae*)	(71.19 ± 0.73)a
Strawberry root rot (*Fusarium oxysporum*)	(70.76 ± 1.32)a
Northern corn leaf blight (*Exserohilum turcicum*)	(51.87 ± 2.86)c

Data are mean ± SE. Different letters in the same column indicate significant difference at the *p* < 0.05 level.

### 3.2. Identification of biocontrol actinomycetes

#### 3.2.1. Morphological identification

**Figure 2:**
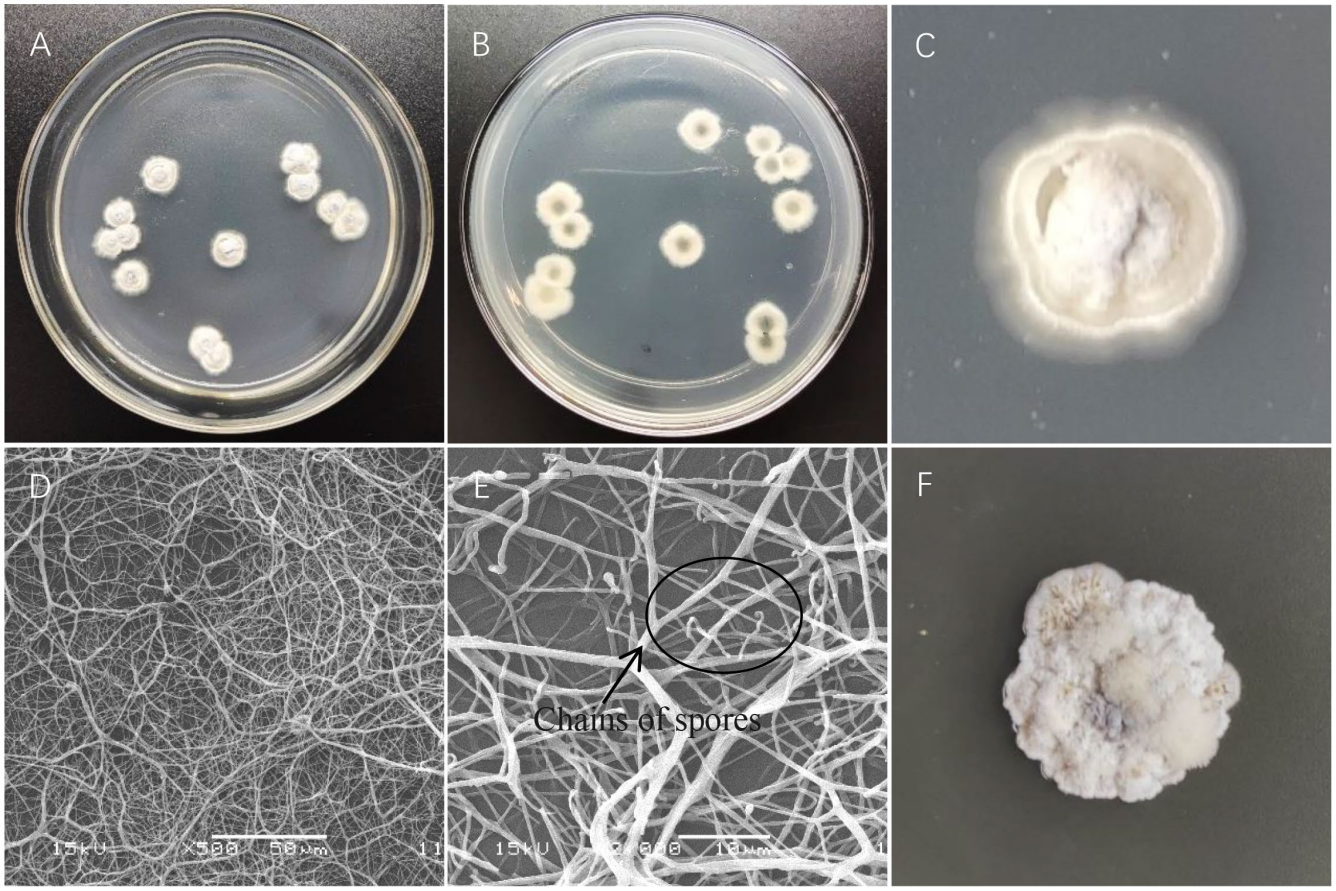
Colony characteristics and microstructural characteristics of *Streptomyces melanosporofaciens* X216. **(A,B)** Colony morphology. **(C)** Growth on Gause’s Synthetic Agar. **(D)** Mycelia. **(E)** Chains of spores. **(F)** Growth on potato dextrose agar.

The colony of strain X216 showed an obvious radial shape when incubated on Gauze’s Synthetic Agar Medium at 28°C. The colony was white and nearly round in the early stage and grew slowly. The mycelia grew radially and densely and tightly in the solid medium. The colony was slightly raised and hard, and the middle of the colony gradually turned black during the later stage and produced a light yellow soluble pigment over time. Strain X216 grew very differently and slightly more quickly on PDA compared with Gause’s Synthetic Agar. The colony was light yellow at first, and it would then produce dense white or light yellow mycelia on the fold. The colony clearly protruded. The colony turned black as it aged and produced a yellow pigment that stained the media light yellow. Coverslips of the insert culture were used for microscopic observation. The mycelia of strain X216 were interwoven, densely connected, lacked septa, and were 0.5–1.1 μm in diameter, and the spore filaments were curved (Figure 2).

#### 3.2.2. Molecular biological identification

**Figure 3:**
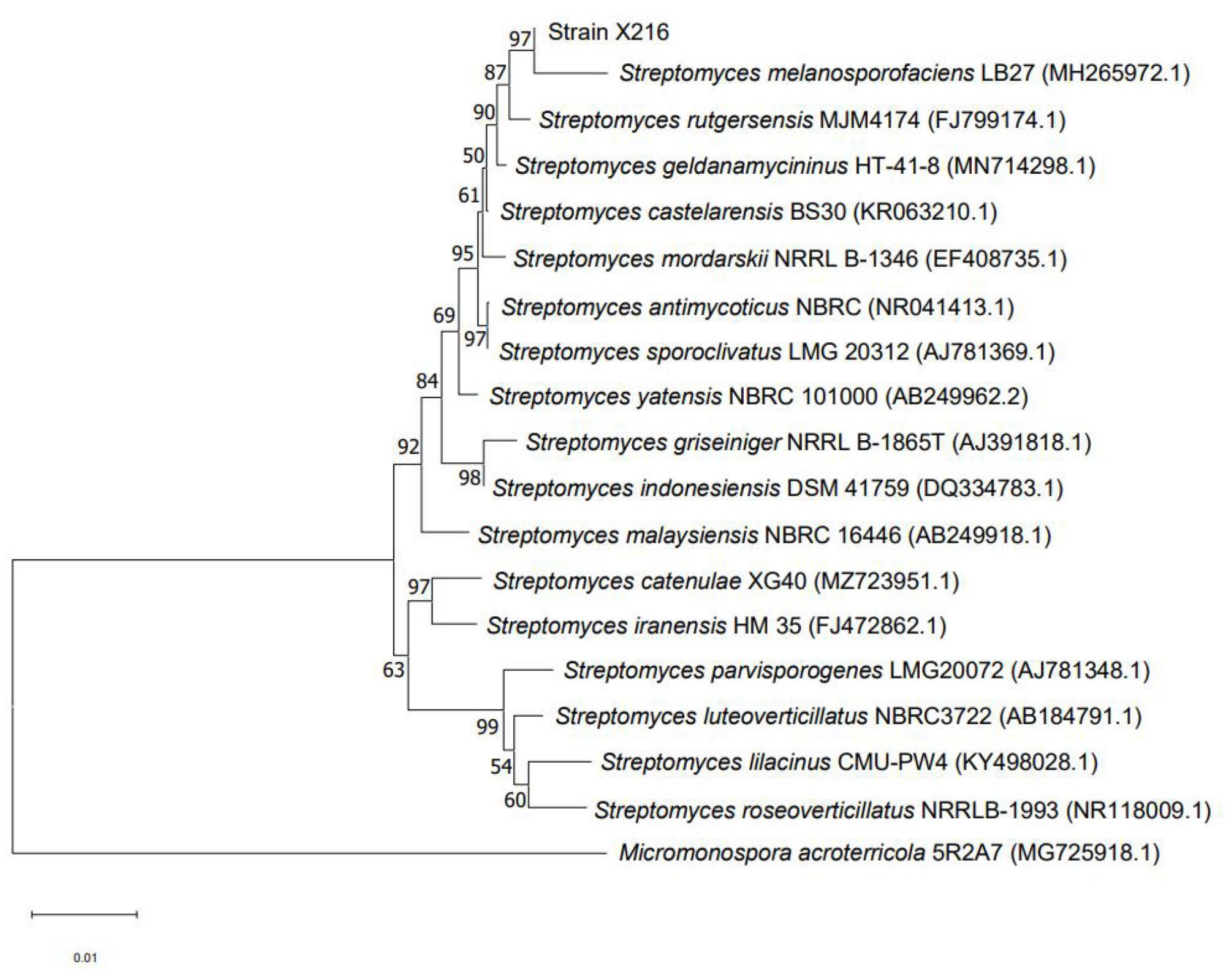
Phylogenetic tree of *Streptomyces melanosporofaciens* X216 based on 16S rRNA gene sequences.

The 16S rRNA gene is the most appropriate indicator to evaluate the phylogeny and classification of bacteria (Zhao et al., 2017). A BLAST analysis on NCBI showed that the sequence of strain X216 was 98.56% homologous to that of *S. melanosporofaciens* (Sequence ID: MH265972.1). MEGA was used to construct a phylogenetic tree, and the closest relative of strain X216 was found to be *S. melanosporofaciens*. The combination of the sequence homology and morphological analysis showed that strain X216 was *S. melanosporofaciens* (Figure 3).

### 3.3. Lethal effect of the actinomycete and different fungicides on the resting spores of *Plasmodiophora brassicae*

**Figure 4:**
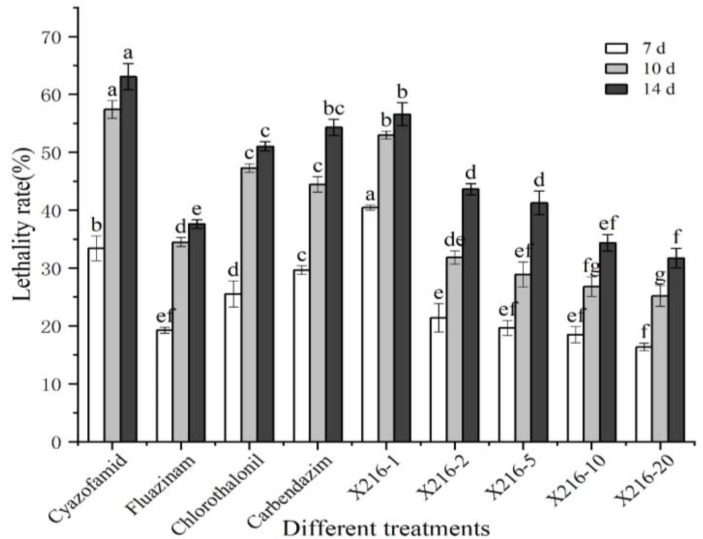
Lethal effect of different treatments on *Plasmodiophora brassicae*. Different letters on the same color bars indicate significant difference at the *p* < 0.05 level.

**Figure 5:**
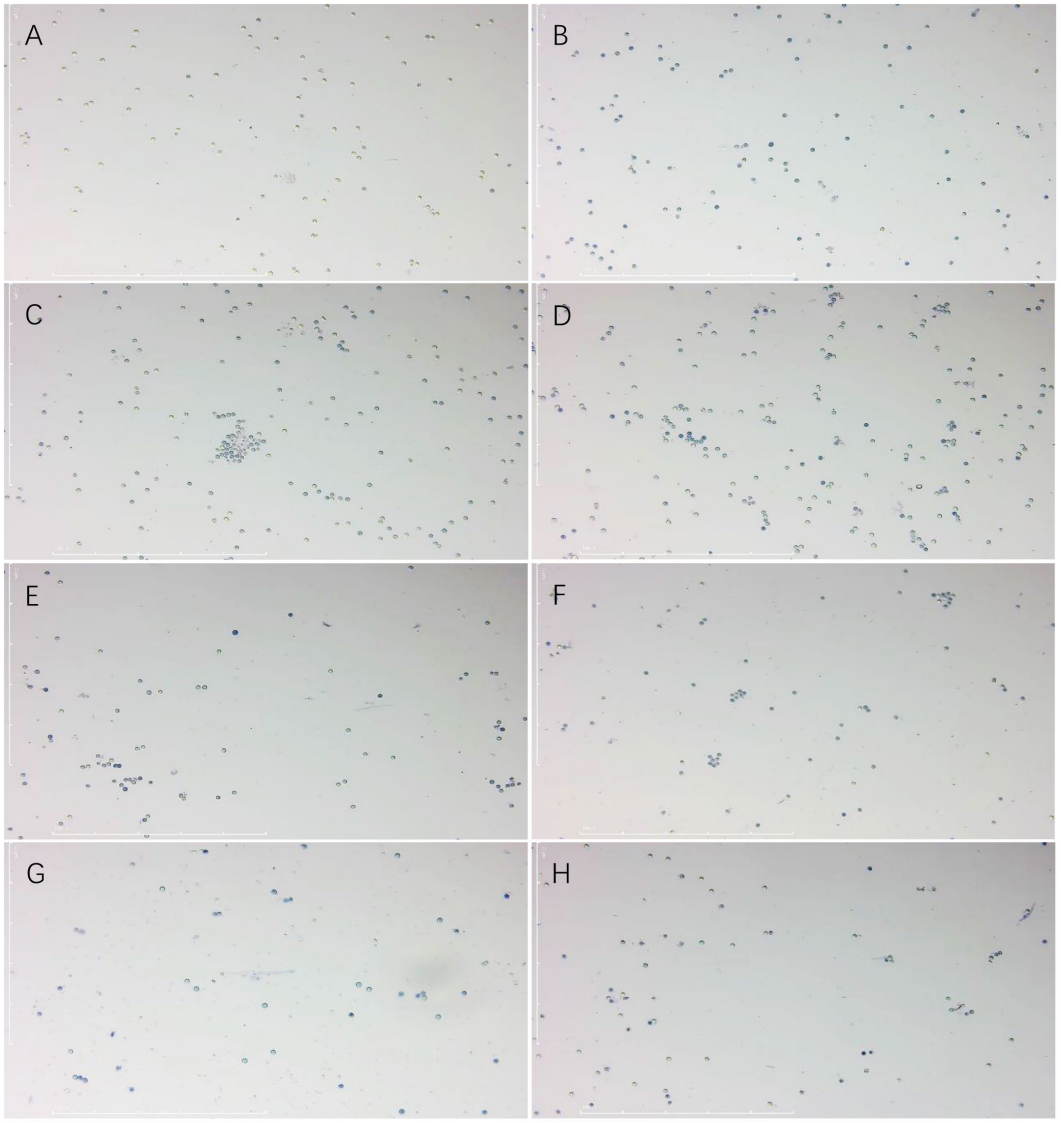
Effect of trypan blue staining on the spores of *Plasmodiophora brassicae*. **(A)** CK. **(B)** 100 g/L cyazofamid SC. **(C)** 20% fluazinam SC. **(D)** 75% chlorothalonil WP. **(E)** 50% carbendazim WP. **(F)** Culture stock solution of *Streptomyces melanosporofaciens* X216. **(G)** Culture of *S. melanosporofaciens* X216 diluted 2-fold. **(H)** Culture of *S. melanosporofaciens* X216 diluted 5-fold.

The results showed that the rate of lethality to the resting spores of *P. brassicae* increased significantly after treatment with a sterile culture solution of strain X216 and several fungicides and the positively correlated with time (Figures 4, 5). The original solution of strain X216 and the culture solution diluted 2-, 5-, 10- and 20-fold were lethal to the resting spores. The corrected lethality rate to the resting spores of the original solution reached 56.59 ± 1.97% after 14 d of treatment, which was not significantly different from that of 50% carbendazim WP during the same period but was significantly higher (*p* < 0.05) than the corrected lethality rate of 75% chlorothalonil WP and 20% fluazinam SC (51.03 ± 0.80 and 37.62% ± 0.72%, respectively). The corrected lethality rate of strain X216 was in the range of 40–45% after 14 d of treatment with cultures diluted by 2- and 5-fold and approximately 30% with cultures diluted by 10- and 20-fold. Among the nine groups of treatments, 100 g/L cyazofamid SC was the most lethal to resting spores with a corrected lethality rate of 63.08% ± 2.28% after 14 d of treatment.

### 3.4. Effect of actinomycetes and different pesticides on the primary infection of *Plasmodiophora brassicae*

**Figure 6:**
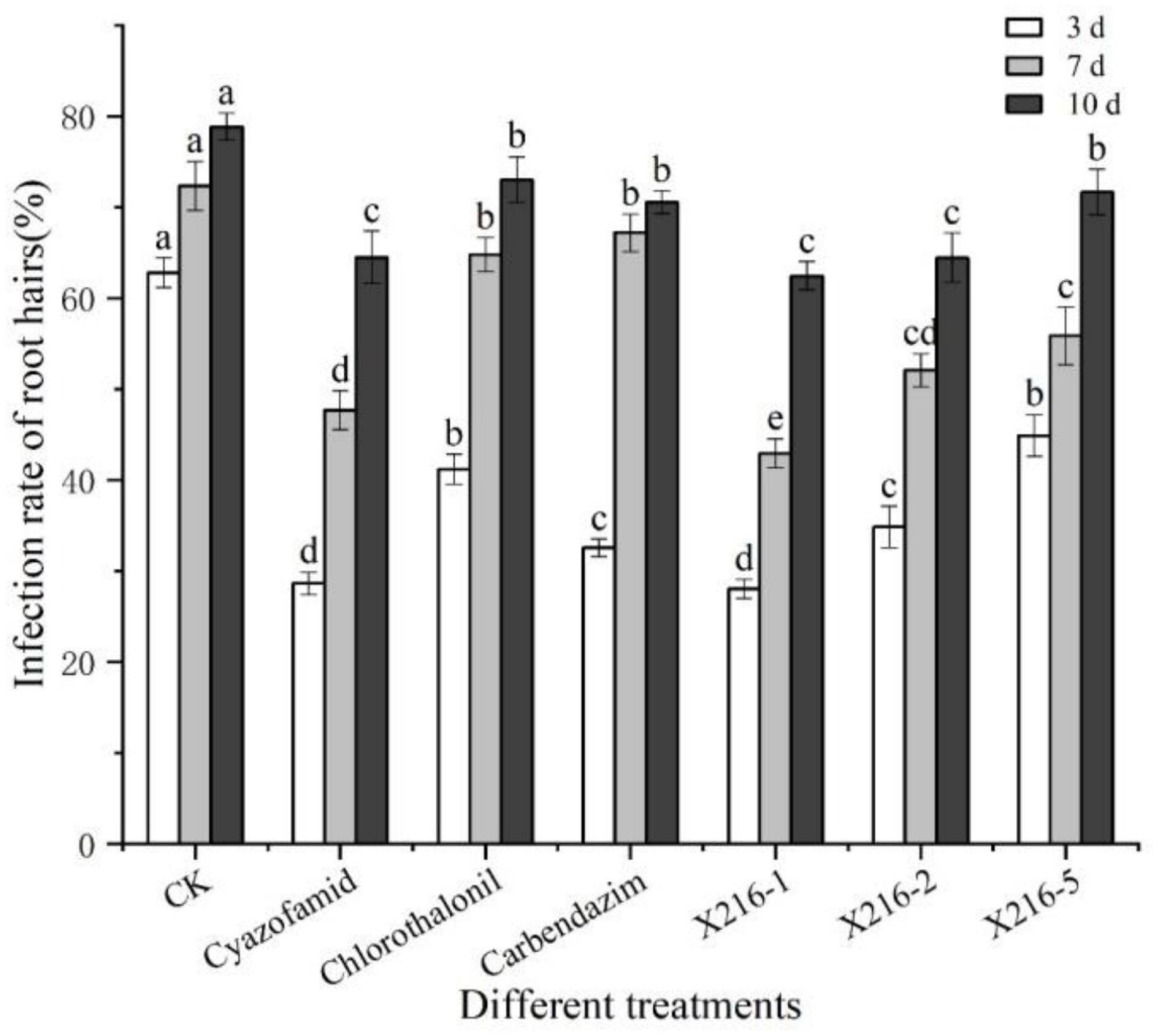
The rate of root hair infection by *Plasmodiophora brassicae* in different treatments at different time points. Different letters on the same color bars indicate significant difference at the *p* < 0.05 level.

**Figure 7:**
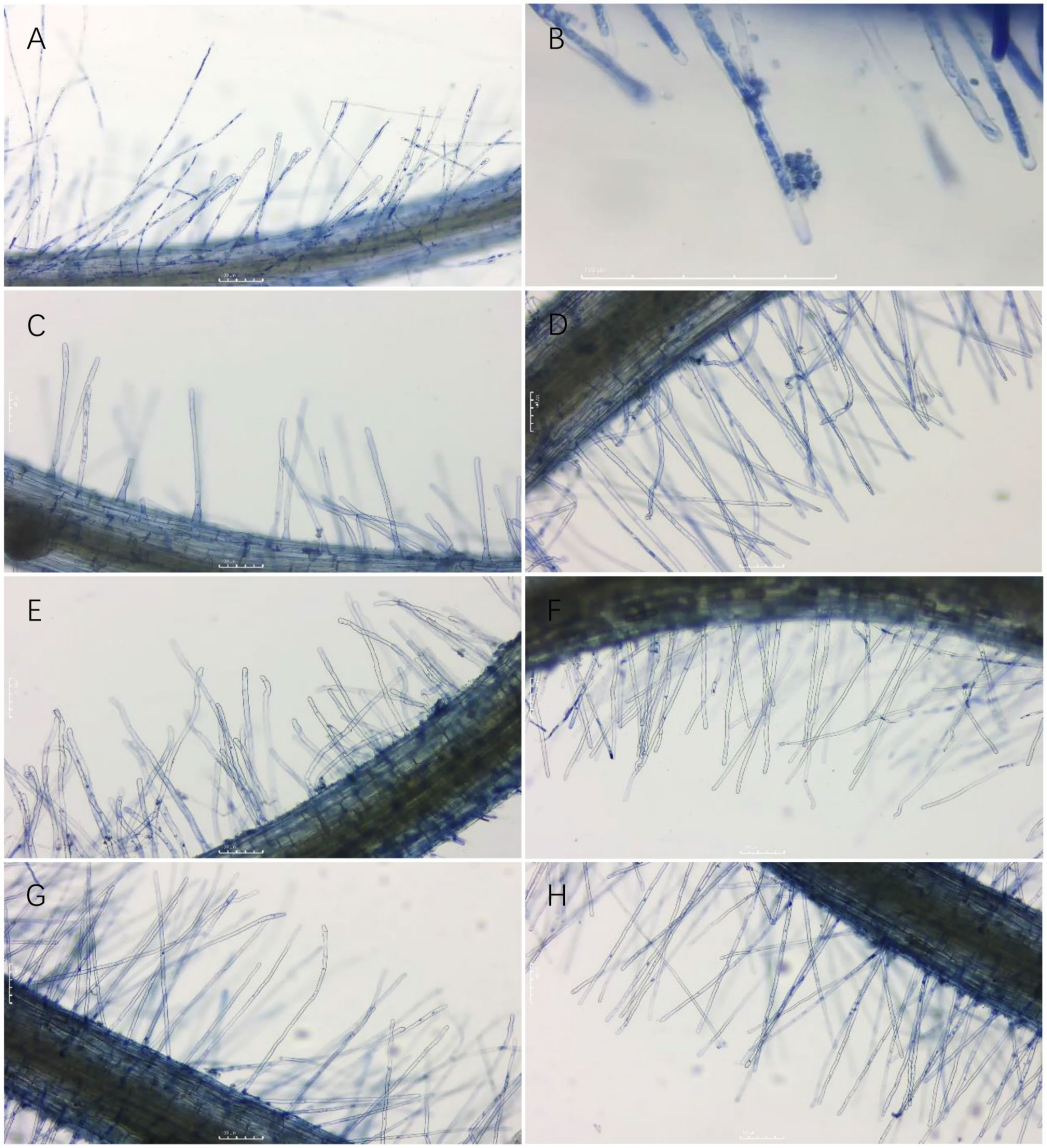
Root hair infection by *Plasmodiophora brassicae* on different treatments three days after infection. **(A,B)**: CK; **(C)**: 100 g/L cyazofamid SC; **(D)**: 75% chlorothalonil WP; **(E)**: 50% carbendazim WP; **(F)**: Strain X216 culture stock solution; **(G)**: Strain X216 culture diluted 2-fold; **(H)**: Strain X216 culture diluted 5-fold.

As shown in Section 3.3, six groups of treatments with 40% or more lethality to resting spores were selected to separately test the effect on primary infection of *P. brassicae* using the rate of infection of root hairs as a reference index. The infected root hairs were dyed dark blue by aniline blue. All six treatment groups were shown to reduce the rate of root hair infection of resting spores of *P. brassicae* (Figures 6, 7). However, the degree of reduction varied. The rate of root hair infection increased continuously with the extension of time, and the control group was found to reach 78.86% ± 1.50% after 10 d. After 10 d of treatment with the sterile culture solution of strain X216, the rate of root hair infection was 62.47% ± 1.55%, which was not significantly different from the infection of 64.49% ± 2.87% treated with 100 g L^−1^ cyazofamid SC during the same period. However, it was significantly lower than that of 75% chlorothalonil and 50% carbendazim WP (73.03 ± 2.51% and 70.56 ± 1.23%, respectively), which reduced the rate of infection of root hairs.

### 3.5. Efficacy of actinomycete and different fungicides on oilseed rape clubroot disease

#### 3.5.1. Efficacy in a pot test

**Figure 8:**
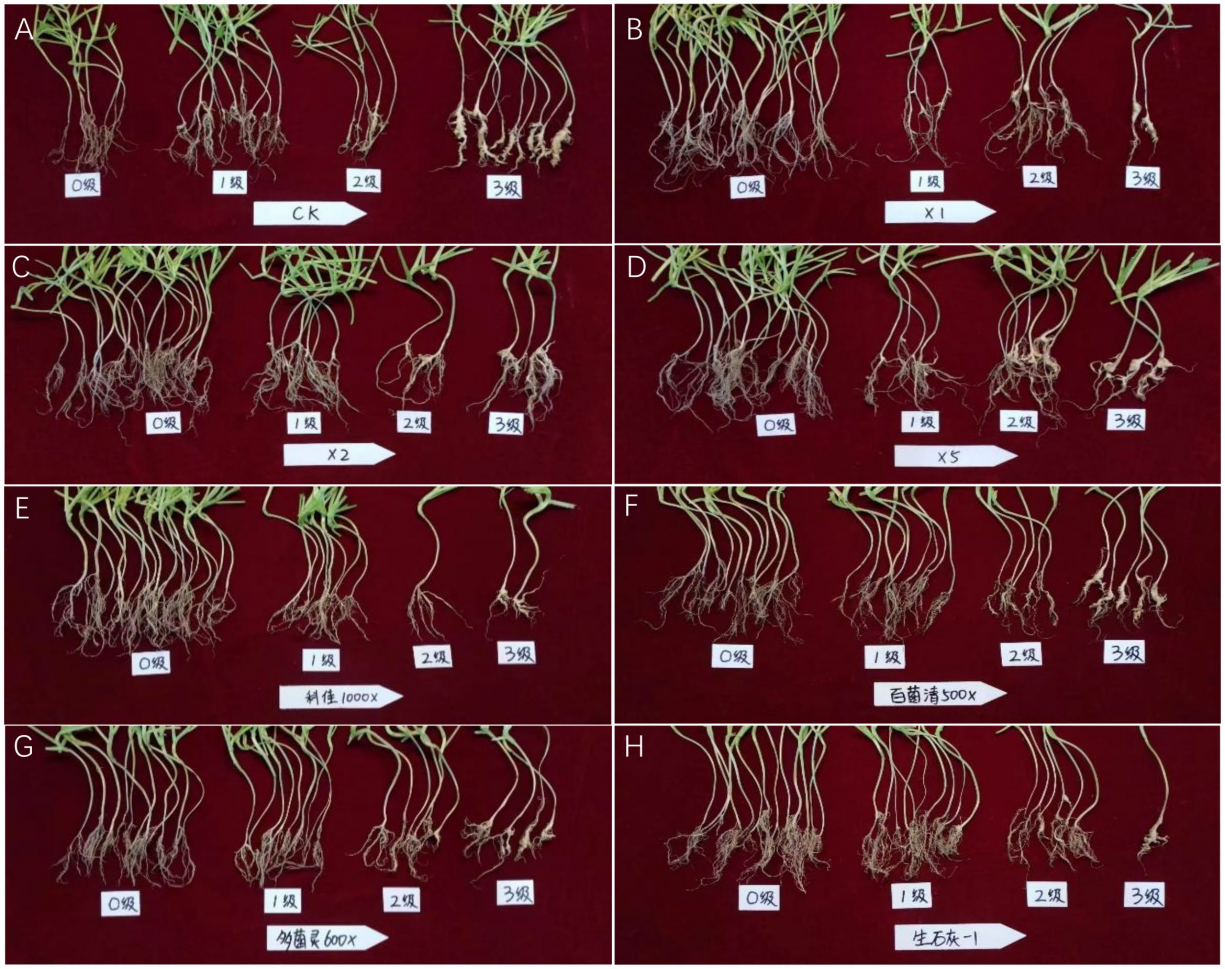
Pot test of different treatments against oilseed rape clubroot. **(A)**: CK; **(B)**: *Streptomyces melanosporofaciens* X216 culture stock solution; **(C)**: *S. melanosporofaciens* X216 culture diluted 2-fold; **(D)**: *S. melanosporofaciens* culture diluted 5-fold; **(E)**: 100 g/L cyazofamid diluted 1,000-fold; **(F)**: 75% chlorothalonil diluted 500-fold; **(G)**: 50% carbendazim diluted 600-fold; **(H)**: 1.2 g calcium oxide per kilogram of soil.

The results of the pot test in the greenhouse showed that the efficacy of the culture of strain X216 and different fungicides on oilseed rape clubroot disease positively correlated with the amount of solution used to irrigate the roots (Table 2; Figure 8). The efficacy of the original solution of strain X216 on the control of oilseed rape clubroot disease was 62.14%, which was not significantly different from that of the chemical fungicide 100 g L^−1^ cyazofamid SC diluted 1,000-fold (67.37%) but was significantly higher (*p* < 0.05) than those of 75% chlorothalonil and 50% carbendazim WP on oilseed rape clubroot disease (26.18 and 27.23%, respectively). When CaO was applied to regulate the soil pH in pots (Zhang et al., 2019), it was the most effective at controlling oilseed rape clubroot disease by 45.52% when 1.2 g of was added to each kilogram of soil.

**Table 2:** Comprehensive control effect of different treatments on oilseed rape clubroot.

Treatment	Dilution ratio	Pot test			Field test		
		Disease incidence/%	Disease index	Control efficiency/%	Disease incidence/%	Disease index	Control efficiency/%
CK	–	(77.17 ± 1.78)a	(51.63 ± 3.30)a	–	(66.87 ± 2.01)a	(58.19 ± 1.32)a	–
X216	1	(32.41 ± 1.45)f	(19.55 ± 0.91)i	(62.14 ± 1.77)a	(42.28 ± 2.59)e	(33.08 ± 0.82)cde	(43.16 ± 1.40)efg
	2	(40.67 ± 2.08)e	(21.81 ± 1.54)hi	(57.76 ± 2.99)ab	(45.57 ± 1.21)de	(35.06 ± 2.55)bcde	(39.75 ± 4.37)efgh
	5	(50.14 ± 2.17)d	(31.58 ± 2.29)fg	(38.84 ± 4.44)d	(53.49 ± 1.81)bc	(40.24 ± 2.27)b	(30.84 ± 3.91)h
100 g/L Cyazofamid SC	1,000	(36.22 ± 3.60)ef	(16.85 ± 0.55)i	(67.37 ± 1.06)a	(20.17 ± 4.62)h	(12.34 ± 2.40)i	(78.79 ± 4.13)a
	1,500	(40.26 ± 1.92)e	(21.36 ± 2.41)hi	(58.62 ± 4.66)ab	(45.15 ± 1.09)de	(21.32 ± 1.75)h	(63.36 ± 3.00)b
	2000	(53.03 ± 2.63)d	(26.30 ± 1.86)gh	(49.07 ± 3.59)bc	(53.60 ± 1.17)bc	(33.02 ± 2.18)cde	(43.25 ± 3.75)efg
75% Chlorothalonil WP	500	(65.75 ± 1.61)c	(38.11 ± 2.03)cd	(26.18 ± 3.92)ef	(30.34 ± 3.03)g	(25.28 ± 3.14)fgh	(56.55 ± 5.40)bcd
	1,000	(64.60 ± 3.58)c	(42.03 ± 0.80)bc	(18.60 ± 1.54)fg	(42.86 ± 2.03)e	(33.14 ± 2.54)cde	(43.05 ± 4.37)efg
	1,500	(75.21 ± 4.29)ab	(46.18 ± 1.92)b	(10.55 ± 3.71)g	(57.62 ± 1.41)b	(37.03 ± 1.72)bcd	(36.36 ± 2.96)fgh
50% Carbendazim WP	600	(64.05 ± 0.96)c	(37.33 ± 3.03)cde	(27.23 ± 6.69)ef	(44.92 ± 1.09)de	(24.15 ± 1.59)gh	(58.50 ± 2.73)bc
	800	(69.36 ± 4.45)bc	(39.01 ± 1.66)c	(24.44 ± 3.22)f	(43.54 ± 3.31)e	(30.68 ± 4.41)def	(47.12 ± 7.81)def
	1,000	(73.25 ± 2.91)ab	(45.70 ± 3.10)b	(11.49 ± 6.01)g	(51.34 ± 3.67)cd	(33.80 ± 0.85)cde	(41.91 ± 1.47)efgh
Calcium oxide (per kilogram of soil)	1.2	(56.07 ± 0.75)d	(28.13 ± 1.47)fg	(45.52 ± 2.84)cd	(35.97 ± 2.34)f	(29.24 ± 3.33)efg	(49.75 ± 5.72)cde
	1.4	(56.48 ± 1.90)d	(32.37 ± 2.60)ef	(37.31 ± 5.04)d	(47.22 ± 0.93)de	(30.50 ± 2.14)def	(47.58 ± 3.67)def
	1.6	(54.80 ± 2.74)d	(33.40 ± 2.08)def	(35.30 ± 4.04)de	(50.17 ± 1.62)cd	(38.88 ± 1.84)bc	(33.19 ± 3.16)gh

Data are mean ± SE. Different letters in the same column indicate significant difference at the *p* < 0.05 level.

#### 3.5.2. Efficacy in the field test

**Figure 9:**
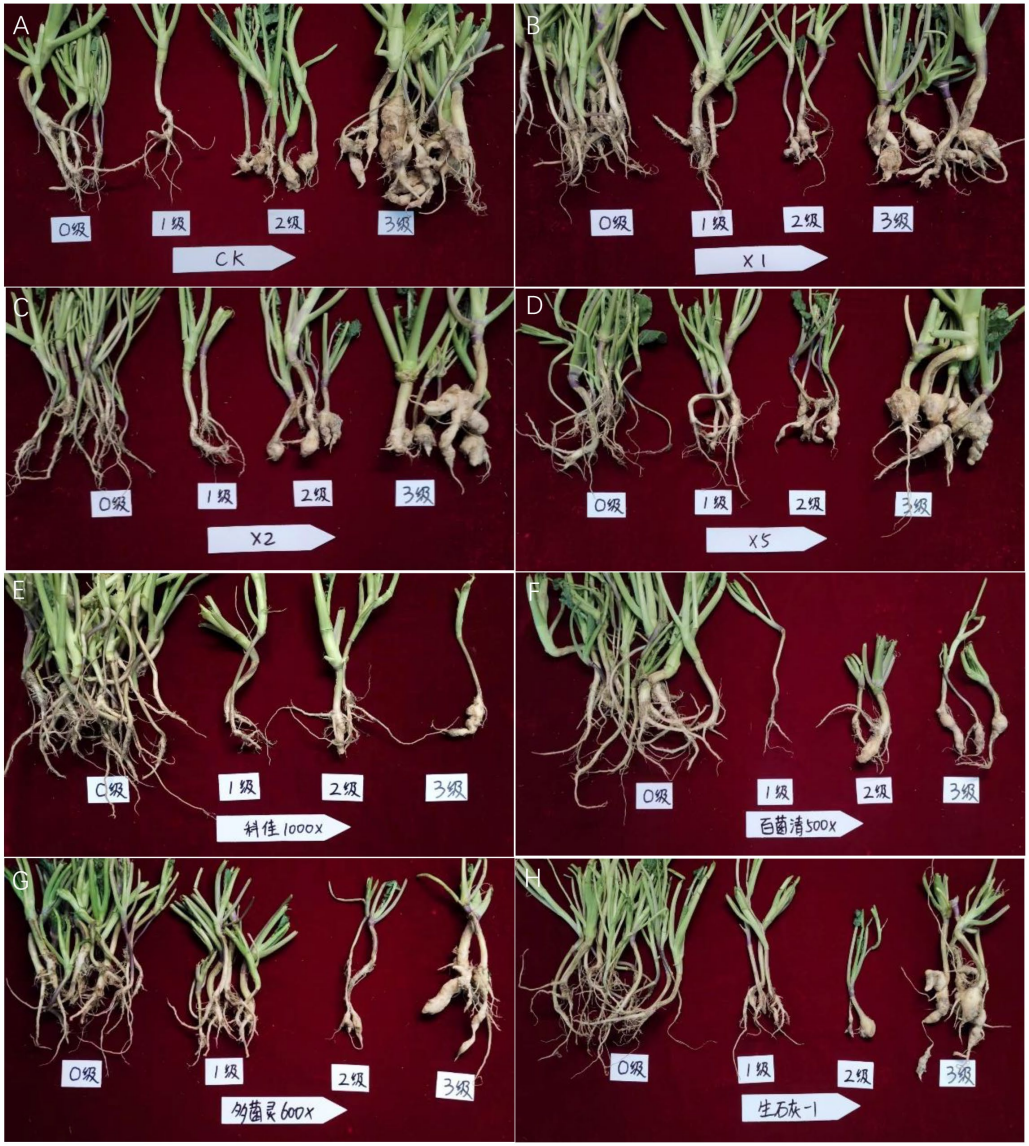
Field test of different treatments against oilseed rape clubroot. **(A)**: CK; **(B)**: *Streptomyces melanosporofaciens* X216 culture stock solution; **(C)**: *S. melanosporofaciens* X216 culture diluted 2-fold; **(D)**: *S. melanosporofaciens* culture diluted 5-fold; **(E)**: 100 g/L cyazofamid diluted 1,000-fold; **(F)**: 75% chlorothalonil diluted 500-fold; **(G)**: 50% carbendazim diluted 600-fold; **(H)**: 1.2 g calcium oxide per kilogram of soil.

The results of the field tests showed that the incidence of oilseed rape clubroot disease was lower in the natural field environment than in the pots (Table 2; Figure 9), which could be owing to the fact that the distribution of resting spores of *P. brassicae* within the field soil was not as uniform as in the potted plants (He et al., 2019). The use of X216 in oilseed rape fields could significantly reduce the disease index of clubroot disease in oilseed rape fields. A solution of strain X216 controlled oilseed rape clubroot disease by 43.16% in the field, which was comparable to that of 100 g L^−1^ cyazofamid SC diluted 2,000-fold and 75% chlorothalonil WP and 50% carbendazim WP diluted 1,000-fold. As the dilution of the culture of strain X216 increased, it became less effective at controlling clubroot disease in the field. There was no significant difference between the control of disease when 75% chlorothalonil WP or 50% carbendazim WP was applied.

#### 3.5.3. Comprehensive analysis of efficacy

A comprehensive comparison of the results of the greenhouse pot test and the field test showed that strain X216 has the ability to control clubroot disease (Table 2). The control effect of the original solution of strain X216 on oilseed rape clubroot disease in the pot test was 62.14%, and the control effect in the field was 43.16%, making it a good biocontrol resource. The control efficiency of strain X216 in the pot test and in the field test were significantly different, which may be related to the inoculum density, the difference in climatic conditions between the greenhouse and the field, and the abundance of the microbial community. The low topography of the experimental field may produce some degree of waterlogging in case of heavy rainfall, which may affect the concentration and colonization of strain X216. The intensity and duration of light in the field are also not as controllable as in the greenhouse, which may affect the actinomycete to exert its control efficiency. In the soil of the pot test there were *P. brassicae* and the actinomycete, but in the field there were many other soil microorganisms in addition to these two, and competitive relationships between the microorganisms may also have contributed to the differences in efficacy.

Control efficiency of the chemical fungicide 100 g L^−1^ cyazofamid SC diluted 1,000-fold was the most stable and effective both in the pot test and field test (67.37 and 78.79%, respectively). The efficacy of 75% chlorothalonil and 50% carbendazim WP in the field test was higher than that in the pot test. The reason may be because they are effective against a wide range of pathogens, inhibiting the damage of a wide range of pathogens on oilseed rape in the field, in order to prevent the decline of the defense of oilseed rape and aggravate the disease index of clubroot disease. As a soil conditioner, calcium oxide was effective against clubroot disease in both greenhouse pot test and the field test.

## 4. Discussion

Since *P. brassicae* grows exclusively in the root cells of the host and still cannot be cultured artificially (Harding et al., 2019), resting spores germinate and infect root hairs as a prerequisite for the disease. This makes them a useful proxy for the amount of *P. brassicae* in the soil. Therefore, this study was conducted to quickly test whether strain X216 has the potential to control oilseed rape clubroot disease. The lethality of the antagonistic strain X216 on the resting spores of *P. brassicae* and its ability to reduce the infection rate of root hairs were determined. The rate of lethality of the resting spores was 56.59 ± 1.97% following the application of a solution of sterile culture stock of strain X216. This was significantly higher than that following treatment with 75% chlorothalonil WP or 20% fluazinam SC. The rate of root hair infection after treatment with a sterile culture stock solution of strain X216 was significantly lower than that of root hair infection following treatment with 75% chlorothalonil and 50% carbendazim WP in the same period.

In this study, the efficacy of the original culture solution of strain X216 against oilseed rape clubroot disease in greenhouse pots was 62.14%, which was better than the field control efficacy of 43.16%. This degree of difference was consistent with the greenhouse control efficacy of *Streptomyces griseoruber* A316 (73.69%) reported by Wang et al. (2012), which was 65.91% higher than the field control. The efficacy of 100 g/L cyazofamid SC diluted 1,000-fold was stable in both the pot and field tests with inhibition by 67.37 and 78.79%, respectively. In contrast, the efficacy of 75% chlorothalonil and 50% carbendazim WP at three dilutions in the pot was lower than that in the field. The reasons for the difference in the efficacy of potted greenhouse control and field control could be owing to the presence of large numbers of microorganisms in the field soil (Zhang Y. et al., 2022) and the existence of competitive microbial relationships; differences in soil properties, such as density, moisture, and organic matter; differences in environmental conditions for growth and development, such as weather and light hours, which are often more complex in the field than in the greenhouse.

The resting spores of *P. brassicae* can proliferate and remain dormant in the soil for a long time, which poses a threat to the production of oilseed rape and increases the difficulty of controlling oilseed rape clubroot disease. There are currently few germplasm resources of oilseed rape that are resistant to clubroot disease, and Huang et al. (2019) reported that 94.74% of more than 200 oilseed rape varieties are susceptible to pathotype 4 of *P. brassicae*. Beneficial microorganisms can be used as biological control agents to control the spread of the soilborne plant diseases (Guo et al., 2020), and this type of biological control is sustainable, green, and environmentally friendly. Thus, it provides a better solution for the control of oilseed rape clubroot disease than the use of fungicides and merits further study.

## Data availability statement

The data presented in the study are deposited in the National Center for Biotechnology Information repository, accession number OR214994.

## Author contributions

Z-hR, LD, and HZo designed the experiment. LD, H-dL, and HZa performed the experiment. Z-hR, LT, and X-jC contributed reagents or materials. LD, HZo, and Z-hR wrote the paper. All authors contributed to the article and approved the submitted version.

## Funding

This work was funded by the Youth Fund of the National Natural Science Foundation of China (32102286), Excellent youth funding of Hunan Provincial Education Department (20B301), General project of Hunan Provincial Education Department (22C0093) and Hunan Provincial Science and Technology Innovation Program (2023NK2024).

## Conflict of interest

The authors declare that the research was conducted in the absence of any commercial or financial relationships that could be construed as a potential conflict of interest.

## Publisher’s note

All claims expressed in this article are solely those of the authors and do not necessarily represent those of their affiliated organizations, or those of the publisher, the editors and the reviewers. Any product that may be evaluated in this article, or claim that may be made by its manufacturer, is not guaranteed or endorsed by the publisher.
